# The Role of Polyphenols in Human Health and Food Systems: A Mini-Review

**DOI:** 10.3389/fnut.2018.00087

**Published:** 2018-09-21

**Authors:** Hannah Cory, Simone Passarelli, John Szeto, Martha Tamez, Josiemer Mattei

**Affiliations:** ^1^Department of Nutrition, Harvard T. H. Chan School of Public Health, Boston, MA, United States; ^2^Massachusetts General Hospital, Charlestown, MA, United States

**Keywords:** polyphenols, antioxidants, chronic disease, food systems, functional foods

## Abstract

This narrative mini- review summarizes current knowledge of the role of polyphenols in health outcomes—and non-communicable diseases specifically—and discusses the implications of this evidence for public health, and for future directions for public health practice, policy, and research. The publications cited originate mainly from animal models and feeding experiments, as well as human cohort and case-control studies. Hypothesized protective effects of polyphenols in acute and chronic diseases, including obesity, neurodegenerative diseases, type 2 diabetes, and cardiovascular diseases, are evaluated. Potential harmful effects of some polyphenols are also considered, counterbalanced with the limited evidence of harm in the research literature. Recent international governmental regulations are discussed, as the safety and health claims of only a few specific polyphenolic compounds have been officially sanctioned. The implications of food processing on the bioavailability of polyphenols are also assessed, in addition to the health claims and marketing of polyphenols as a functional food. Finally, this mini-review asserts the need for increased regulation and guidelines for polyphenol consumption and supplementation in order to ensure consumers remain safe and informed about polyphenols.

## Introduction

Polyphenols, organic compounds found abundantly in plants, have become an emerging field of interest in nutrition in recent decades. A growing body of research indicates that polyphenol consumption may play a vital role in health through the regulation of metabolism, weight, chronic disease, and cell proliferation. Over 8,000 polyphenols have thus far been identified, though their short- and long-term health effects have not been fully characterized (1). Animal, human and epidemiologic studies show that various polyphenols have antioxidant and anti-inflammatory properties that could have preventive and/or therapeutic effects for cardiovascular disease, neurodegenerative disorders, cancer, and obesity (2, 3). However, some have cautioned that there may be harmful effects of overconsumption, especially in cases where compounds are isolated rather than consumed in a food matrix (4, 5). This narrative mini-review explores the current evidence relating polyphenols to general health and non-communicable diseases (NCDs), describes the implications of this evidence for public health, and discusses potential future directions for practice, policy, and research.

## Scientific background

### General evidence on health

Recent polyphenol research is primarily composed of epidemiologic cohort and case-control studies that focus on disease endpoints, in addition to mouse-model and human feeding experiments that explore mechanistic interactions. Evidence generated by animal and human studies shows that the antioxidant and anti-inflammatory properties of polyphenols may potentially prevent or serve as treatment against many non-communicable diseases (Table 1) (2, 3).

**Table 1:** Summary of Reviewed Potential Health Effects of Polyphenols.

**Type of disease**	**Evidence of effects**
Neurodegenerative diseases	Curcumin, resveratrol, and catechins (like epigallocatechin gallate (EGCG)) may protect against Alzheimer's-like diseases and dementia through antioxidant and immunomodulatory and scavenging properties that protect neurons and inhibition of the neurotoxic effects of the beta-amyloid protein, the accumulation of which is linked to Alzheimer's disease (6–15). The iron-chelating effects of EGCG, curcumin, myricetin, ginsenosides, and ginkgetin are thought to be an underlying mechanism through which polyphenols prevent neurotoxicity, leading to a neuroprotective effect against neurodegenerative diseases like Parkinson's Disease, Alzheimer's Disease, and Huntington's Disease (16, 17).
Inflammation	Phenolic compounds may prevent systemic and/or localized inflammation by restoring the redox balance to reduce oxidative stress, and by modulating inflammatory responses through mitigation of cytokine pathways (14).
Cancer	Flavanoids such as anthocyanins, catechins, flavanols, flavones, flavanones, and isoflavones, may neutralize free radicals and decrease cancer risk by arresting cellular growth in tumors (18). Specific types of cancers with evidence of beneficial effects from polyphenols include colon, prostate, epithelial, endometrial, and breast cancer (14, 15, 19–22).
Cardiovascular health	Flavonoid-rich foods have been associated with improved ventricular health, reduced platelet activity, enzymatic modulation, anti-inflammatory effects, and lower blood pressure, to increase overall vascular health (15, 23). Flavonoids and resveratrol may block cholesterol oxidation to reduce LDL and lower risk of cardiovascular disease (14, 20, 21, 24–27).
Type 2 diabetes	Several polyphenolic compounds, anthocyanins being the most substantiated, are associated with both the prevention and management of type 2 diabetes through protection of beta cells from glucose toxicity, anti-inflammatory and antioxidant effects, slowing of starch digestion, and regulation and altered transport of glucose, leading to better glycemic control (6, 28–30).
Obesity	Polyphenols like catechins, resveratrol, and curcumin are associated with anti-obesogenic effects, potentially through adipocyte oxidation, inhibition of lipogenesis, reduction in inflammation, and increases in energy expenditure, leading to improved weight loss and maintenance (18). A number of polyphenols have been shown to have protein-binding properties that can inhibit starch, lipid, and protein digestion in the gastrointestinal tract by interacting with and inhibiting digestive enzymes (23, 28–32).

Current literature suggests that the long-term consumption of diets rich in polyphenols protects against certain cancers, cardiovascular diseases, type 2 diabetes, osteoporosis, pancreatitis, gastrointestinal problems, lung damage, and neurodegenerative diseases (19, 28, 33, 34). The dominant explanation for these benefits is the “biochemical scavenger theory,” which posits that polyphenolic compounds negate free radicals by forming stabilized chemical complexes, thus preventing further reactions (35). There is also evidence of an additional mechanism by which polyphenols protect against oxidative stress by producing hydrogen peroxide (H_2_0_2_), which can then help to regulate immune response actions, like cellular growth (35, 36). Yet, the majority of evidence comes from *in vitro* models and it is unclear if these mechanisms hold true in humans (37–40). Furthermore, recent evidence has elucidated the effect of absorption pharmacokinetics on efficacy of polyphenols as antioxidants and other potentially health-promoting mechanisms; these physiochemical properties of the molecules may explain the variable effects observed in human and animal models, as well as conflicting data in the literature (41–45).

Some harmful effects have been reported from polyphenol intake. Adverse outcomes have been documented from polyphenolic botanical extracts in beverages, especially for individuals with degenerative disease, high blood pressure, thyroid disease, epilepsy, or heart disease (46). Due to pre-absorptive interactions during digestion, dietary polyphenols have also been shown to reduce the transport of thiamin and folic acid, and to alter the activity of drugs through interactions that affect drug transporters or enzymes involved in reactions, resulting in both inhibition and increasing bioavailability depending on the case (14). For example, the iron-chelating and inhibitory effects on absorption of iron associated with polyphenols may lead to poor iron status (47). This could be harmful for populations consuming crops rich in phytates that also inhibit iron absorption, such as sorghum, beans, and millet, especially for populations that already have marginal iron stores. Isoflavones may impact the long-term growth and pubertal development of children fed soy-based formulas in infancy (48, 49). Previous research suggested that isoflavones, found in soy products, may adversely affect women with or at-risk for estrogen-sensitive breast cancer and endometrial cancer as a result of the endocrine-disrupting properties of these compounds (50, 51); however, recent epidemiological reviews suggest either a null or protective effect of isoflavones on these cancer types (52, 53). A recent report by the European Food Safety Authority found no risk of taking isoflavone-containing food supplements for peri- and post-menopausal women (54).

### Non-communicable diseases

Evidence suggests that polyphenols inhibit pro-inflammatory transcription factors by interacting with proteins involved in gene expression and cell signaling, leading to protective effects against many inflammation-mediated chronic diseases (55). Polyphenols hypothesized to be anti-carcinogenic are thought to arrest cellular growth by inducing cell senescence or apoptotic cell death, and their differential redox status may selectively affect tumor cells (14). Resveratrol, found in red wine, is reported to prevent platelet aggregation and relax the arterial blood vessels, disrupting the oxidation of low density lipoprotein (LDL) cholesterol (20, 21, 24–26). However, a systematic review and meta-analysis of 282 human studies found that resveratrol supplementation had no impact on blood lipid levels. This may be because resveratrol is usually consumed in small quantities, and thus any protective effect is marginal (27).

Anthocyanins have been associated with both the prevention and management of type 2 diabetes in animal, human, and epidemiological studies (28). The mechanisms of these benefits vary based on the polyphenolic compound, but include protection of pancreatic beta cells from oxidation, anti-inflammatory and antioxidant action, decreased starch digestion due to the suppression of enzyme activity, and the inhibition of advanced glycation end product formation (28). A number of studies have shown improved fasting glucose levels, and improved glucose tolerance and insulin sensitivity, with the consumption of foods containing anthocyanins (56).

### Neurodegenerative diseases

Some polyphenols may also protect against neurodegenerative diseases, including Alzheimer's, Parkinson's, and Huntington's diseases. A population-based prospective study in the Bordeaux region found that consuming three to four glasses of wine per day, which contains resveratrol, was associated with an 80% lower incidence of dementia and Alzheimer's disease compared to non-drinkers (6). Other epidemiologic evidence from the Copenhagen City Heart Study has shown that monthly or weekly red wine was associated with a reduced risk of neurodegenerative diseases, while the other alcoholic beverages studied, beer and spirits, were not (7). Animals studies have shown that a class of polyphenols, epigallocatechin gallate (EGCG), competitively inhibited a neurotoxin known to induce Parkinson's-like disease (8). EGCG may also protect neurons by activating cell survival signaling pathways (9). Turmeric, which is found in curry and contains the polyphenol curcumin, has been hypothesized to contribute to the low incidence of Alzheimer's disease in India due to its high rate of consumption. Improved cognitive function have been found in a study of elderly South Asian participants who frequently consumed curry compared to those who rarely did (10). A prospective cohort study found that Japanese elderly adults who drank green tea had a lower incidence of cognitive decline compared to non-tea drinkers and compared to coffee and black tea drinkers, after adjusting for other factors like alcohol consumption and physical activity (11).

### Obesity

Numerous cellular and animal, and some human studies, have examined the impact of polyphenols on weight status. In the cases of population-based studies, the reduced risk of obesity associated with polyphenol intake from foods may be confounded by the fact that polyphenolic-rich foods are nutrient-dense rather than energy-dense, resulting in a lower calorie intake overall. Evidence from *in-vitro* and randomized-controlled trials suggest that certain polyphenolic compounds promote a reduction in the genesis, differentiation, and proliferation of adipocytes, in addition to the prevention of inflammation and promotion of lipolysis (18).

Catechin polyphenols, including EGCG, have been associated with antioxidant, anti-inflammatory and anti-mutagenic effects (57–59). Catechins are thought to prevent weight gain by promoting greater energy expenditure and fat oxidation (60), though evidence suggests that the effects of green tea on fat oxidation may be due to an interaction with caffeine consumption (59). A meta-analysis of 11 randomized trials found that participants randomized to green tea consumption were better able to maintain their weight loss compared to non-green tea drinkers (61).

Blueberries, a rich source of anthocyanins, reduce weight gain in animal studies by protecting against inflammation and modulating obesity pathways (62). Yet, animal trials have demonstrated mixed effects for mitigating weight gain depending on the form in which anthocyanins were consumed (63).

Findings from studies investigating the anti-obesogenic properties of resveratrol in animal and human studies have been mixed. Resveratrol has been shown to inhibit lipogenesis and adipocyte differentiation in rats (64). A systematic review and meta-analysis of 282 human subjects found that resveratrol supplementation had no impact on blood triglyceride levels (65). Because resveratrol is consumed in small quantities, its protective effects are unlikely with standard levels of intake (56).

Lastly, curcumin has been hypothesized to reduce adiposity through increased energy metabolism, reduced inflammation, and suppressed angiogenesis (66). Animal studies have shown that supplementation with dietary curcumin led to reductions in adiposity and liver fatty acid synthesis, and higher fatty acid oxidation levels (67).

### Interaction with gut microbiota

A growing area of interest in the field of polyphenols is their potential interactions with gut microbiota. Though the mechanisms are not entirely understood, it is hypothesized that polyphenol metabolites may promote beneficial gut bacteria, while inhibiting invasive species (68, 69). Trials have found that blueberry extract drink can promote presence of beneficial bifidobacteria (70), while green tea extract has been found to modulate bacteria like *Clostridium difficile, Escherichia coli*, and *Salmonella typhimurium* (71, 72). Evidence suggests that these interactions may also modulate potential impacts on chronic disease risk, such as improved insulin sensitivity and the atheroprotective and hepatoprotective effects of polyphenols (73–77). There is growing evidence that the presence of phenolic compounds may promote beneficial actions of probiotics (78). Further research is needed to characterize the bioavailability of polyphenols and how related metabolites, either phase II metabolites or those generated from gut microbiota, may interact with systemic tissues, in both *in vitro* and *in vivo* models (79–81).

## Implications for food systems and policy

### Food processing

Food processing and storage strongly influences the polyphenol content of foods. Certain compounds are prone to oxidation, and the addition of polyphenols to foods may compromise shelf stability (82, 83) Methods to prevent this are currently being researched (84). In other cases, antioxidants derived from fruits, vegetables, mushrooms, and herbs have been used to inhibit lipid and protein oxidation and prevent microbial activity in meats (84, 85). Food manufacturers and processors take advantage of the antioxidant properties of polyphenols by adding them to foods and drinks, such as meats and beer, so that they can be sacrificed to prevent the oxidation of other compounds in the food, like lipids, to increase shelf stability (86–91). As use of these extracts potentially broadens, the antioxidant polyphenols they contain could lead to meats becoming a source of dietary polyphenols (92, 93).

Culinary preparation plays a significant role in polyphenol content. The quercetin content of tomatoes and onions can be reduced by up to 80% from boiling, 65% from microwaving, and 30% from frying (24). Other types of antioxidants have shown the opposite trend; carotenoids were most depleted in frying methods and most retained from boiling methods in carrots, zucchini, and broccoli, although polyphenolic content was highest when these vegetables were raw and depleted with any cooking (94). Overall, the relationship between cooking method and polyphenol availability is complex and depends on the food, polyphenolic compound, cooking method, and other factors, often exhibiting a U-shaped relationship.

Food processing also impacts the bioavailability of polyphenols. Removal of peels and hulls can strip foods of their polyphenol content, while maceration can facilitate the diffusion of polyphenols. For example, red wine is produced through maceration with polyphenol-rich grape skins, resulting in a polyphenol content 10-times greater than white wine (95). Processing methods of foods, like fermentation and drying, can promote the production of toxic substances, including biogenic amines, which has been shown to be counteracted by some polyphenols (91, 96–99).

### Marketing and regulation

Due to the many findings of health benefits, many strategies have emerged to market polyphenols to consumers. Polyphenol-containing products are being promoted as functional foods, which are “foods that have a potentially positive effect on health beyond basic nutrition” (4). The global polyphenols market, which includes applications in food and beverages, pharmaceuticals, and cosmetics, was estimated to exceed 700 million USD in 2015 and is projected to reach 1.1 billion USD by 2022 (99, 100). Some research has shown improvements in biomarkers—including glycemic response (82)—from polyphenols consumed in these forms, but their long-term effects have not been fully assessed. The ability to evaluate and market polyphenol products is also growing, with new methods and procedures currently being developed for assessing bioavailability and bioaccessibility in different foods (101, 102). While it is unlikely to be able to catalog all of the effects of polyphenols per dose due to the myriad of compounds, their interactions with other compounds during consumption, and our incomplete understanding of their effects on health, these methods are important to begin to understand safe levels of consumption.

No regulatory recommendations currently exist for the consumption of polyphenols in functional foods (5). Creating such regulations is challenging due to the multitude of compounds, the limited evidence from human studies, and variability in the polyphenol content of foods. Furthermore, the lack of standardized methods, cost of analysis, shelf instability, and lack of intake references make it difficult to add information on food polyphenol content to labels (103). The United States Food and Drug Administration allows health claims for antioxidant nutrients with an established Recommended Daily Intake (RDI), for example, vitamins A and C. Because polyphenols are neither a vitamin nor have an RDI, they cannot be marketed with health claims (104). Polyphenols are often sold as nutritional supplements, which are minimally regulated in the United States, meaning a greater number of functional claims can be made (104).

A danger of under-regulated supplementation is the risk of creating mega-doses of polyphenols. The risks of polyphenol intake are difficult to quantify, as the majority of studies investigating risk have been *in vitro*. Despite myriad studies highlighting potential benefits, unambiguous links between polyphenols and human health have been few and far between. This gap exists largely due to the difficulty of mimicking *in vivo* conditions effectively in *in vitro* models. At this time, the European Food Safety Authority only permits health claims for olive oil hydroxytyrosol and cocoa flavanols (81, 105, 106). The health effects of mega-doses of polyphenolic compounds are unlikely to be feasibly characterized by research and as such, alternative approaches must be developed to understand the efficacy of compound-containing foods and supplements and guide regulation efforts. A 2014 report found that 20% of drug-related liver injuries were due to herbal and dietary supplements, many of which contain polyphenols (107). Green tea extract supplements are commonly marketed for weight loss; however, high doses of catechins found in green tea have been found to cause hepatotoxicity, possibly due to oxidative stress caused by EGCG and its metabolites (108, 109). The current lack of regulation in the United States may contribute to overhyped claims, potentially resulting in misuse and overconsumption at potentially harmful levels by consumers (110).

### Concerns regarding polyphenol fortification and supplementation

There are some concerns regarding polyphenol fortification and supplementation. First, their consumption may replace intake of healthy whole foods, like fruits and vegetables. Moreover, polyphenol extracts used in supplementation and fortification may lack the synergistic effects and health benefits of a diet naturally rich in polyphenols (111). These additional benefits include consumption of a high-fiber diet, intake of other and potentially interacting nutrients and non-nutrients, and satiation. In polyphenol research, it is challenging to understand the complex interactions underlying the functional benefits observed with consumption of whole foods containing polyphenols (4). Consumption of the isolated polyphenolic compounds alone may not produce the same benefits observed in epidemiological studies, or the benefits may be overstated by food marketing companies.

Fortified foods may also be more energy-dense, rather than nutrient-dense, which could offset any potential anti-obesogenic effects of polyphenols and potentially lead to weight gain (5). Cellular and animal trials test for benefits of polyphenols at amounts much higher than those commonly found in human diets, thus the level at which they can be safely and beneficially added to foods for human consumption remains unclear.

The potential for the consumption of deleterious levels of polyphenols is especially of concern with supplements. Some manufacturers recommend intakes over 100-times higher than those currently associated with a Western diet (110). In some cases, supplementation trials of antioxidants have been associated with adverse effects, including increased mortality or stroke in some studies (112–115). Concerns regarding heterogeneous effects in subpopulations and interactions with medications also arise with the promotion of polyphenol consumption at levels far above natural occurrence (114).

Without a complete understanding of the safe and beneficial levels of polyphenol intake, their fortification in foods cannot be adequately informed. Researchers should be extremely cautious before undertaking supplementation trials of polyphenolic compounds in humans to ensure that mechanisms and effects *in vivo* are well-understood.

## Conclusions

This narrative mini-review provides an overview of the role of polyphenols in relation to topics highly that are relevant to nutrition research and practice, including obesity, type 2 diabetes, neurodegenerative diseases, and gut microbiota. There is substantial evidence that specific polyphenols benefit health status, especially for the prevention and management of certain chronic diseases. The ability to harness these benefits is limited by the current understanding of mechanisms, dosage requirements, and potential unintended effects. Potential negative outcomes for some subgroups should be investigated, and additional human studies are needed to confirm biological mechanisms and public health implications of polyphenols. Studies *in vitro* and in animals have used levels much higher than those commonly found in human diets, and so the level at which polyphenols can be safely and beneficially consumed remains unclear. Further research is needed to understand whether and how the same benefits from polyphenols consumed in whole foods can be derived from isolated forms.

The multitude of polyphenols with different structures, pathways, and physiological roles makes it challenging to fully elucidate their short and long term health effects. As scientific understanding of polyphenols grows, consumers' awareness of proposed benefits and potential risks will increase, as will marketing efforts and the need for understanding efficacy to guide regulation. Regulatory bodies should consider staying abreast of the scientific evidence to provide guidelines for polyphenol consumption and supplementation, including the regulation of their health and functional claims, and the establishment of Dietary Reference Intakes (DRIs) for common and/or potentially harmful polyphenols. Because polyphenols are most commonly found in healthful, plant-based foods like fruits and vegetables, recommendations for consumption should be tied into existing nutrition education efforts and guidelines to promote healthy diets. Although much remains unknown in this burgeoning field, public health measures should be taken early to ensure that consumers are safe and informed.

## Author contributions

HC and SP conceptualized the topic, researched and analyzed the literature, and wrote the manuscript, including interpretations. JS analyzed background literature and drafted portions of the manuscript. MT and JM provided substantial scholarly guidance on the conception of the topic, manuscript draft and interpretation, and revised the manuscript critically for intellectual content. All authors approve the final version of the manuscript, ensure the accuracy and integrity of the work, and agree to be accountable for all aspects of the work.

### Conflict of interest statement

The authors declare that the research was conducted in the absence of any commercial or financial relationships that could be construed as a potential conflict of interest.
